# Evaluating the potential impact and cost-effectiveness of dapivirine vaginal ring pre-exposure prophylaxis for HIV prevention

**DOI:** 10.1371/journal.pone.0218710

**Published:** 2019-06-26

**Authors:** Meghan Reidy, Elizabeth Gardiner, Carel Pretorius, Robert Glaubius, Kristine Torjesen, Katharine Kripke

**Affiliations:** 1 Avenir Health, Washington, District of Columbia, United States of America; 2 AVAC, New York, New York, United States of America; 3 Avenir Health, Glastonbury, Connecticut, United States of America; 4 FHI 360, Durham, North Carolina, United States of America; Kenya Medical Research Institute, UNITED STATES

## Abstract

**Background:**

Expanded HIV prevention options are needed to increase uptake of HIV prevention among women, especially in generalized epidemics. As the dapivirine vaginal ring moves forward through regulatory review and open-label extension studies, the potential public health impact and cost-effectiveness of this new prevention method are not fully known. We used mathematical modeling to explore the impact and cost-effectiveness of the ring in different implementation scenarios alongside scale-up of other HIV prevention interventions. Given the knowledge gaps about key factors influencing the ring’s implementation, including potential uptake and delivery costs, we engaged in a stakeholder consultation process to elicit plausible parameter ranges and explored scenarios to identify the possible range of impact, cost, and cost-effectiveness.

**Methods and findings:**

We used the Goals model to simulate scenarios of oral and ring pre-exposure prophylaxis (PrEP) implementation among female sex workers and among other women ≤21 years or >21 years with multiple male partners, in Kenya, South Africa, Uganda, and Zimbabwe. In these scenarios, we varied antiretroviral therapy (ART) coverage, dapivirine ring coverage and ring effectiveness (encompassing efficacy and adherence) by risk group. Following discussions with stakeholders, the maximum level of PrEP coverage (oral and/or ring) considered in each country was equal to modern contraception use minus condom use in the two age groups.

We assessed results for 18 years, from 2018 to 2035. In South Africa, for example, the HIV infections averted by PrEP (ring plus oral PrEP) ranged from 310,000 under the highest-impact scenario (including ART held constant at 2017 levels, high ring coverage, and 85% ring effectiveness) to 55,000 under the lowest-impact scenario (including ART reaching the UNAIDS 90-90-90 targets by 2020, low ring coverage, and 30% ring effectiveness). This represented a range of 6.4% to 2.2% of new HIV infections averted. Given our assumptions, the addition of the ring results in 11% to 132% more impact than oral PrEP alone. The cost per HIV infection averted for the ring ranged from US$13,000 to US$121,000.

**Conclusions:**

This analysis offers a wide range of scenarios given the considerable uncertainty over ring uptake, consistency of use, and effectiveness, as well as HIV testing, prevention, and treatment use over the next two decades. This could help inform donors and implementers as they decide where to allocate resources in order to maximize the impact of the dapivirine ring in light of funding and implementation constraints. Better understanding of the cost and potential uptake of the intervention would improve our ability to estimate its cost-effectiveness and assess where it can have the most impact.

## Introduction

Despite successes in scaling up antiretroviral therapy (ART) in many countries, which can help reduce HIV transmission when viral load suppression is achieved, there are still as many as 1.8 million new HIV infections annually. In sub-Saharan Africa, women account for more than half of all new adult HIV infections [1]. Access to and use of daily oral pre-exposure prophylaxis (PrEP) has been increasing in sub-Saharan Africa since September 2015, when the World Health Organization (WHO) issued guidance recommending daily oral PrEP for people at “substantial risk” of HIV infection (defined by the WHO as HIV incidence greater than 3% in the absence of daily oral PrEP) [2]. Yet not everyone at substantial risk will be able to effectively use oral PrEP. Prevention gaps will remain, which could be addressed by offering more biomedical prevention choices, similar to how expanded method mix has led to greater uptake in the contraceptive field [3]. Additional biomedical prevention approaches are under development in an effort to provide a suite of HIV prevention options that can be used effectively by a wider subset of the population at risk. The monthly dapivirine vaginal ring is one of these potential prevention options, which specifically addresses the need for a woman-centered product. Developed by the International Partnership for Microbicides, the dapivirine ring is a flexible, silicone ring that provides sustained release of the antiretroviral (ARV) drug dapivirine over one month to reduce the risk of HIV-1 acquisition. Phase III clinical trials (i.e., ASPIRE and The Ring Study) showed that the ring reduced HIV infection by approximately 30 percent overall [4]. Post-hoc exploratory analyses suggested that HIV risk was reduced by up to 75% among a subset of participants who appeared to have better adherence [5]. As these results are influenced by challenges in quantifying adherence due to measurement error of drug levels in the ring, the effectiveness of the ring at near-perfect adherence may be even higher. Further data are anticipated from two recently completed open-label extension (OLE) studies, HOPE and DREAM, in which ring was provided to previous Phase III trial participants. Preliminary findings from these two studies suggest higher overall levels of adherence, leading to higher effectiveness (estimated to be around 50% using modeling), than were seen during the Phase III trials, similar to the experience with oral PrEP in Phase III versus OLE [6,7,8].

As the ring moves forward through regulatory review, the potential impact and cost-effectiveness of this new HIV prevention product are not fully known. There are significant knowledge gaps with regard to the ring’s potential uptake, delivery costs, and effectiveness in real-world settings. This study used mathematical modeling to explore dapivirine ring impact and cost-effectiveness in different implementation scenarios, alongside scale-up of HIV treatment and other prevention interventions, in order to define the range of potential impact.

Given the knowledge gaps related to rollout of the ring, we conducted a stakeholder consultation process to elicit plausible parameter ranges, and we explored different scenarios to bookend impact, cost, and cost-effectiveness. Forty-four stakeholders representing donors, United Nations agencies, product developers, modelers, advocates, and implementers were engaged in consultations individually, in small groups, or a large group in one case (with 37 participants). Stakeholder consultation respondents offered a wide, and often divergent, range of opinions. Stakeholders recommended that modeling include countries where the ring clinical trials had been held and that represented a range of epidemic contexts. Recommendations related to estimates of coverage—meaning the percentage of women in each risk group with oral PrEP pills or the ring in their possession at a given point—ranged from a minimum of 5% to 10%, to a maximum of 20% to 30%, and there were disparate opinions of whether contraceptive prevalence was an appropriate benchmark. Regarding subpopulations, there was a consensus that although younger women generally have higher HIV incidence than older cohorts, younger women may have lower uptake, and this should be included in modeling, although age disaggregation is challenging due to paucity of data. Respondents had trouble quantifying potential adherence to the ring. Efficacy estimates were highly divergent, from 90% to 95% down to 30%. The results of the stakeholder consultation emphasized the degree to which we do not yet know what the actual values of these parameters will be in non-research settings.

## Methods

### Model

We used Goals, a dynamic, compartmental HIV epidemic model within the Spectrum suite of models (developed by Avenir Health [9]), to simulate scenarios of PrEP implementation in Kenya, South Africa, Uganda, and Zimbabwe among medium-risk women less than or equal to 21 years of age and greater than 21 years, as well as female sex workers (FSWs). Here, we use the term “PrEP” to refer to the general class of products for preventing HIV prior to exposure using ARV drugs, regardless of delivery modality (i.e., oral, ring, injectable, implant). Goals simulates transmission of HIV and its morbidity and mortality consequences for adult populations ages 15 to 49 years, which are structured into five mutually exclusive risk categories: 1) low risk (stable couples, defined as men and women reporting a single sexual partner in the last year), 2) medium risk (men and women with more than one partner in the last year), 3) high risk (FSWs and their male clients), 4) men who have sex with men, and 5) male and female people who inject drugs.

Medium-risk women 21 years of age or less and greater than 21 years are embedded within the medium-risk group in Goals. The Goals model was modified to accept correction factors based on the relative population size and relative incidence within each age group by country. These correction factors are derived from the AIDS Impact Module (AIM) in Spectrum, which Goals uses to disaggregate the impact between the two age groups. National AIM files are publicly available in Spectrum and are updated and validated annually by Ministry of Health staff in each country in a process coordinated by the Joint United Nations Programme on HIV/AIDS (UNAIDS) to produce national, regional, and global estimates of HIV burden.

In various test scenarios, we varied ART coverage, oral PrEP and dapivirine ring coverage, and ring effectiveness (encompassing efficacy and adherence) by risk group. In the Goals model, we used the average coverage in a year to represent the entire year. Unless otherwise stated, all scenarios used the following assumptions: moderate oral PrEP coverage (level varied by country and risk group, see Table 1), moderate ring coverage (level varied by country and risk group, see Table 1), oral PrEP effectiveness of 71%, ring effectiveness of 50% [6,7], and ART scale-up achieving the 90-90-90 targets by 2020 [10]. Condom use and voluntary medical male circumcision (VMMC) rates were held constant at 2017 levels for all scenarios. See S1 Table for additional details of all scenarios described below.

**Table 1:** PrEP (oral or ring) coverage values by risk group and country.

	Kenya	Zimbabwe	Uganda	South Africa
**High**				
Female sex workers	36%	36%	36%	36%
Medium-risk women >21	30%	37%	15%	26%
Medium-risk women ≤ 21	15%	18%	14%	25%
**Moderate**				
Female sex workers	18%	18%	18%	18%
Medium-risk women >21	15%	18%	7%	13%
Medium-risk women ≤ 21	8%	9%	7%	12%
**Low**				
Female sex workers	9%	9%	9%	9%
Medium-risk women >21	8%	9%	4%	7%
Medium-risk women ≤ 21	4%	5%	3%	6%

### ART scale-up scenarios

We evaluated four different ART scenarios:

1. Base scenario, achieving 90-90-90 by 2020 (Scenario a): Country achieves the UNAIDS 90-90-90 targets by 2020, which means that 90% of all people living with HIV (PLHIV) know their HIV status, 90% of those who know their status (81% of PLHIV) are on ART, and 90% of ART patients (73% of PLHIV) are virally suppressed. This assumption was used except where otherwise indicated.
2. Low ART scenario, continuing current coverage (Scenario b): ART and viral suppression coverages remain at 2017 levels.
3. Intermediate scenario 1, 90-90-90 by 2030 (Scenario c): Country achieves 90-90-90 targets by 2030.
4. Intermediate scenario 2, 90-90-90 by 2020 among women only (Scenario d): Country achieves 90-90-90 targets by 2020, but only for women, while men remain at 2017 levels.

### Ring coverage scenarios

We developed seven scenarios of ring scale-up involving different combinations of high, moderate, low, and no ring scale-up in the three risk groups, with maximum ring coverage levels being reached in 2035 (with scale-up beginning in 2023). These seven ring scale-up scenarios were conducted in the context of moderate oral PrEP coverage for all populations, with maximum oral PrEP coverage reached in 2030 (with scale-up beginning in 2018), and used a moderate level of ring effectiveness (50%). A counterfactual of no ring or oral PrEP coverage was used to calculate the number of HIV infections averted by PrEP (oral plus ring).

The combined PrEP (oral plus ring) reference coverage for the model’s medium risk group was set at the level of modern contraception use among sexually active women (minus the level of condom use for the purpose of family planning) in each of the two age groups [11,12,13,14]. We benchmarked PrEP coverage to the use of modern contraception as a proxy for health system access and capacity, as well as the ability and motivation of women in a given context to use a prevention intervention. Our expert consultations advised that this was a reasonable reference in the absence of real-world uptake data on the ring.

For FSWs, the combined PrEP reference coverage was set at 60%. The coverage of 30% per method for FSWs was informed by oral PrEP uptake in demonstration projects in South Africa and Benin [15,16]. See Table 1 for coverage values by risk group in each country. High coverage for each method was 60% of the reference coverage level for each risk group/country; moderate coverage was 30%, and low coverage was 15%, such that the total combined PrEP coverage would be 120% of the reference if both methods had high coverage, 60% if both had moderate coverage, and 30% if both had low coverage. It should be noted that because the high and medium risk groups are only a fraction of the total female population, the coverage of PrEP in the overall population is much lower than the indicated value.

In addition to the scenarios where all risk groups achieved the same level of coverage, we evaluated three scenarios where coverage varied between risk groups: (5) ring coverage is lower among medium-risk women, (6) ring coverage is lower among younger women (intended to represent a scenario of lower uptake of the ring among younger women, similar to Phase III trial findings indicating lower use and adherence in this group), and (7) no use of the ring among younger women (to represent a case in which the ring is not approved or recommended for use in younger women). See Table 2 for the coverage patterns and Table 3 for an example of the resulting values for South Africa.

**Table 2:** Ring coverage scenarios.

	PrEP Coverage Scenarios					
	Oral PrEP Coverage Patterns			Ring Coverage Patterns		
Scenarios	FSWs	Med >21	Med ≤ 21	FSWs	Med >21	Med ≤ 21
**1. Oral PrEP only (moderate)**	Moderate	Moderate	Moderate	No scale-up	No scale-up	No scale-up
**2. Oral PrEP (moderate) and ring (moderate)**	Moderate	Moderate	Moderate	Moderate	Moderate	Moderate
**3. Oral PrEP (moderate) and ring coverage (low)**	Moderate	Moderate	Moderate	Low	Low	Low
**4. Oral PrEP (moderate) and ring (high)**	Moderate	Moderate	Moderate	High	High	High
**5. Ring coverage lower among medium-risk women**	Moderate	Moderate	Moderate	Moderate	Low	Low
**6. Ring coverage lower among younger women**	Moderate	Moderate	Moderate	Moderate	Moderate	Low
**7. No use of ring among younger women**	Moderate	Moderate	Moderate	Moderate	Moderate	No scale-up

**Table 3:** South Africa PrEP coverage values.

	PrEP Coverage Scenarios						Total PrEP Coverage		
	Oral PrEP Coverage Levels			Ring Coverage Levels					
Scenarios	FSWs	Med >21	Med ≤ 21	FSWs	Med >21	Med ≤ 21	FSWs	Med >21	Med ≤ 21
**1. Oral PrEP only (moderate)**	18%	13%	12%	0%	0%	0%	18%	13%	12%
**2. Oral PrEP (moderate) and ring (moderate)**	18%	13%	12%	18%	13%	12%	36%	26%	25%
**3. Oral PrEP (moderate) and ring (low)**	18%	13%	12%	9%	7%	6%	27%	20%	19%
**4. Oral PrEP (moderate) and ring (high)**	18%	13%	12%	36%	26%	25%	54%	39%	37%
**5. Ring coverage lower among medium-risk women**	18%	13%	12%	18%	7%	6%	36%	20%	19%
**6. Ring coverage lower among younger women**	18%	13%	12%	18%	13%	6%	36%	26%	19%
**7. No use of ring among younger women**	18%	13%	12%	18%	13%	0%	36%	26%	12%

### Ring effectiveness scenarios

We examined low, moderate, and high ring effectiveness in all risk groups, as well as in different combinations, to represent potential variations in adherence in different risk groups. All ring effectiveness scenarios included moderate PrEP coverage (from Scenario 2 in Table 2). These scenarios were: (11) higher ring adherence among FSWs, (12) lower ring adherence among younger women, and (13) both higher ring adherence among FSWs and lower adherence among younger women. Levels ranged from a low of 30% to a high of 85% as a sensitivity analysis around the standard assumption of 50% effectiveness (see Table 4) [4,5,6,7]. For this analysis we chose to not disaggregate efficacy and adherence (the combination of which constitutes effectiveness), but we assumed that adherence (not efficacy) is the mechanism through which effectiveness would vary.

**Table 4:** Ring effectiveness scenario values.

	Ring Effectiveness		
Scenarios	FSWs	Med >21	Med ≤ 21
**9. High ring effectiveness**	0.7	0.7	0.7
**10. Low ring effectiveness**	0.3	0.3	0.3
**11. Higher ring adherence among FSWs**	0.7	0.5	0.5
**12. Lower ring adherence among younger women**	0.5	0.5	0.3
**13. Higher adherence among FSWs + lower adherence among younger women**	0.7	0.5	0.3

### Bookend scenarios

In order to represent the extremes of the range of possible impact, the upper bound was set by combining the most optimistic PrEP coverage and effectiveness with the low-ART scenario (i.e., continue current coverage), while the lower bound was set by combining the most pessimistic PrEP coverage and effectiveness with the base ART scenario (i.e., achieving 90-90-90 by 2020).

### Unit costs

The unit costs for oral PrEP in Kenya and South Africa came from costing studies conducted in each country using comparable approaches and cost categories [17,18]. The Kenya costs were translated to Zimbabwe and Uganda, where unit cost data encompassing the same cost categories were not available in the literature, using the gross national income (GNI) per capita to convert values for cost categories that were driven by labor costs. Costs represent a provider perspective. We assumed the service delivery costs for the ring were the same as those for oral PrEP and that only product and laboratory testing costs varied across countries. For the product component of the ring unit cost, we used a cost of US$7 per ring and 12 rings per year. For the ring, we assumed that HIV testing was the only laboratory test required, whereas for oral PrEP, hepatitis B and creatinine testing were also included. Included in Table 5 is the cost of a person-year of ART in each country, as the incremental cost of PrEP for each scenario takes into account ART cost savings from HIV infections averted by PrEP during the period assessed. All of these costs were held constant over time.

**Table 5:** Total unit cost in US$ for oral PrEP, the ring, and antiretroviral treatment per client per year, by country.

	Oral PrEP	Ring	ART
**Kenya**	$202	$189	$257
**Zimbabwe**	$121	$157	$254
**Uganda**	$133	$154	$445
**South Africa**	$160	$152	$286

## Results

The results for all of the scenarios described above, representing sensitivity analyses around ART scale-up, ring coverage, oral PrEP coverage, and ring effectiveness, are presented in full in S2 Table and the person-years of PrEP coverage in each scenario is shown in S3 Table. A selection of results is presented below.

### What is the range of potential impact of PrEP (oral plus ring) in the most extreme scenarios?

Evaluating the base and low-ART scenarios, combined with the extremes of ring coverage and effectiveness, allows us to bookend the range of highest and lowest potential impact of PrEP (see Table 6, Scenarios 14 and 15). The maximum potential impact in South Africa is far greater in absolute terms than in any other country included in the analysis, with 310,000new HIV infections averted by ring and oral PrEP from 2018–2035. The maximum potential impact in Kenya, Uganda, and Zimbabwe is 64,000, 53,000, and 50,000 new HIV infections averted, respectively. In relative terms, the potential maximum impact ranges from 7.7% (HIV infections averted divided by the total number of HIV infections in the counterfactual scenario) in Kenya to 4.5% in Uganda, based on variations in assumed ring coverage in each country. Comparatively, the minimum potential impact for PrEP is 55,000 new HIV infections averted in South Africa, 10,000 in Kenya, 10,000 in Uganda, and 8,000 in Zimbabwe. In relative terms, the minimum potential impact ranges from 2.3% in Kenya 1.5% in Uganda.

**Table 6:** Range of absolute and relative impact of PrEP (oral plus ring) (2018–2035) in new HIV infections averted.

		Kenya		Uganda		South Africa		Zimbabwe	
		No.	%	No.	%	No.	%	No.	%
**14. Highest ring impact (current ART levels, 85% effectiveness, high ring coverage)**	**Ring**	35,000	4.2%	29,000	2.5%	176,000	3.6%	26,000	3.2%
	**Oral PrEP**	29,000	3.5%	24,000	2.0%	134,000	2.7%	24,000	3.0%
	**Total**	64,000	7.7%	53,000	4.5%	310,000	6.3%	50,000	6.2%
**15. Lowest ring impact** **(90-90-90 by 2020, 30% effectiveness, low ring coverage)**	**Ring**	1,000	0.2%	1,000	0.1%	6,000	0.2%	1,000	0.2%
	**Oral PrEP**	10,000	2.1%	9,000	1.4%	49,000	2.1%	7,000	1.9%
	**Total**	11,000	2.3%	10,000	1.5%	55,000	2.3%	8,000	2.0%

Absolute impact is shown as the number of new HIV infections averted, while relative impact is defined as the number of HIV infections averted by PrEP divided by the total number of HIV infections without PrEP.

### How will different scenarios for coverage of the dapivirine ring affect the impact of the HIV prevention program?

**Fig 1:**
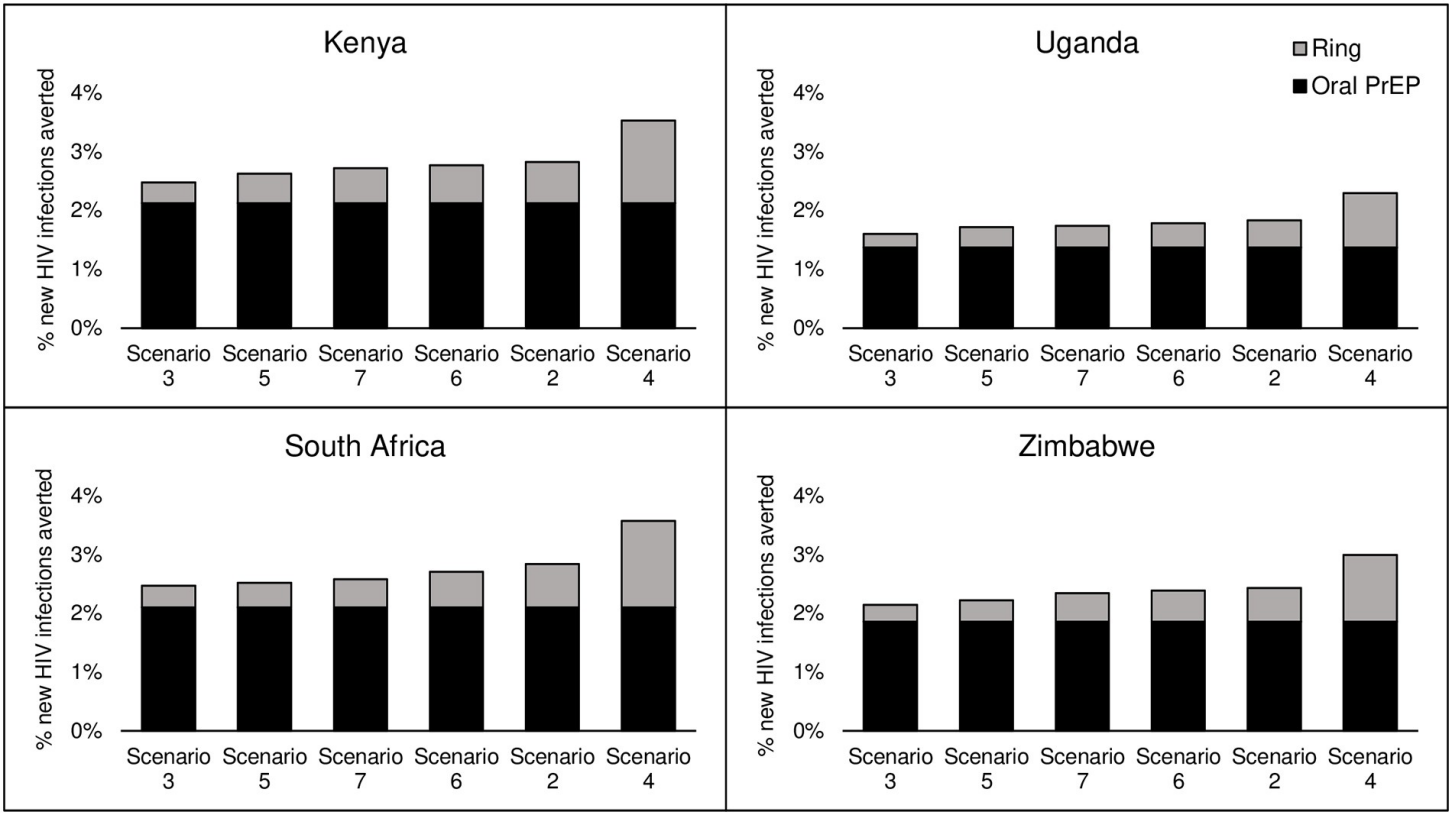
Impact of ring coverage scenarios on the percentage of new HIV infections averted by PrEP (oral plus ring) (2018–2035). Scenarios: (3) Low ring coverage, (5) Ring coverage lower in medium-risk, (7) No ring coverage in women ≤ 21, (6) Lower ring coverage in women ≤ 21, (2) Moderate ring coverage, (4) High ring coverage.

Comparing each ring coverage scenario with our counterfactual scenario with no ring or oral PrEP gives us an estimate of the contribution of the ring to HIV infections averted at different levels of coverage. All scenarios below (Fig 1) assume moderate coverage of oral PrEP and that countries reach 90-90-90 by 2020. As seen previously, in absolute terms, the largest combined impact of ring plus oral PrEP is in South Africa, ranging from 58,000 infections averted in the low-coverage scenario to 84,000 in the high-coverage scenario. Ring impact roughly corresponds to the level of ring coverage in the overall population. However, the relative impact shows some variability between countries, due to the differences in HIV incidence among risk groups, as well as in levels of ring coverage. Almost identical levels of relative impact are seen in Kenya and South Africa for the low-, moderate-, and high ring coverage scenarios.

### How will different scenarios for ART scale-up affect PrEP (oral plus ring) impact?

**Fig 2:**
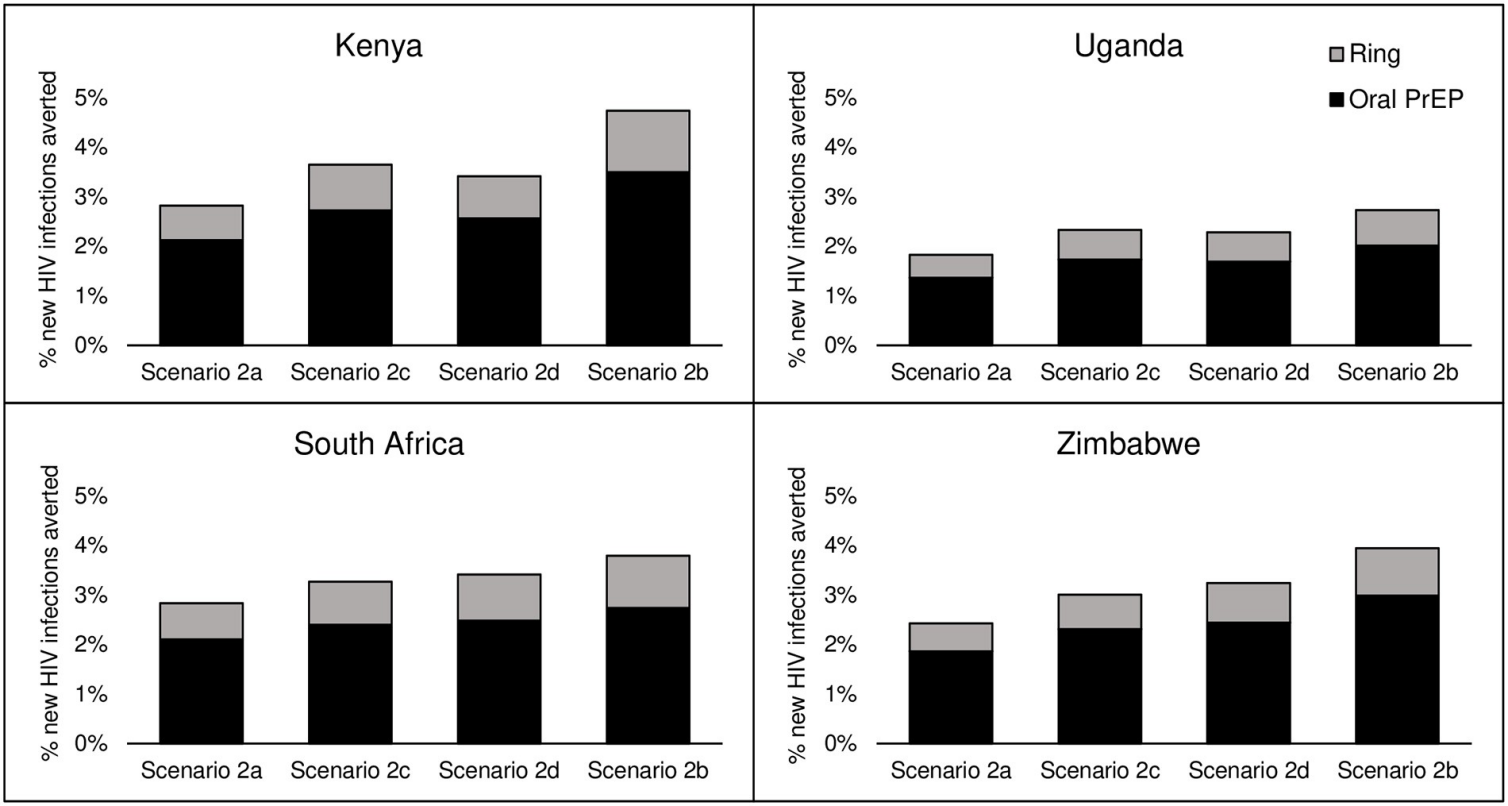
Impact of ART scale-up scenarios on the percentage of new HIV infections averted (2018–2035). Scenarios: (2a) Base scenario, achieving 90-90-90 by 2020; (2c) Intermediate scenario 1, 90-90-90 by 2030; (2d) Intermediate scenario 2, 90-90-90 by 2020 among women only; (2b) Low ART scenario, continuing current coverage.

Using the standard scenario of moderate oral PrEP coverage, moderate ring coverage, and moderate ring effectiveness (Scenario 2) and varying ART scale-up, we see the strong influence treatment as prevention has on the potential impact of PrEP. Our base scenario of achieving 90-90-90 by 2020 (Scenario 2a) allows for the lowest potential impact, ranging from a low of 10,000 new HIV infections averted by PrEP in Zimbabwe to 67,000 in South Africa. The highest impact comes from the low ART scale-up scenario (2b) ranging from 32,000 new infections averted in Zimbabwe to 185,000 in South Africa. The relative impact of the two intermediate ART scale-up scenarios (90-90-90 by 2030 and 90-90-90 by 2020 among women only) varies by country, as seen in Fig 2.

### How will different scenarios for effectiveness of the dapivirine ring affect impact?

**Fig 3:**
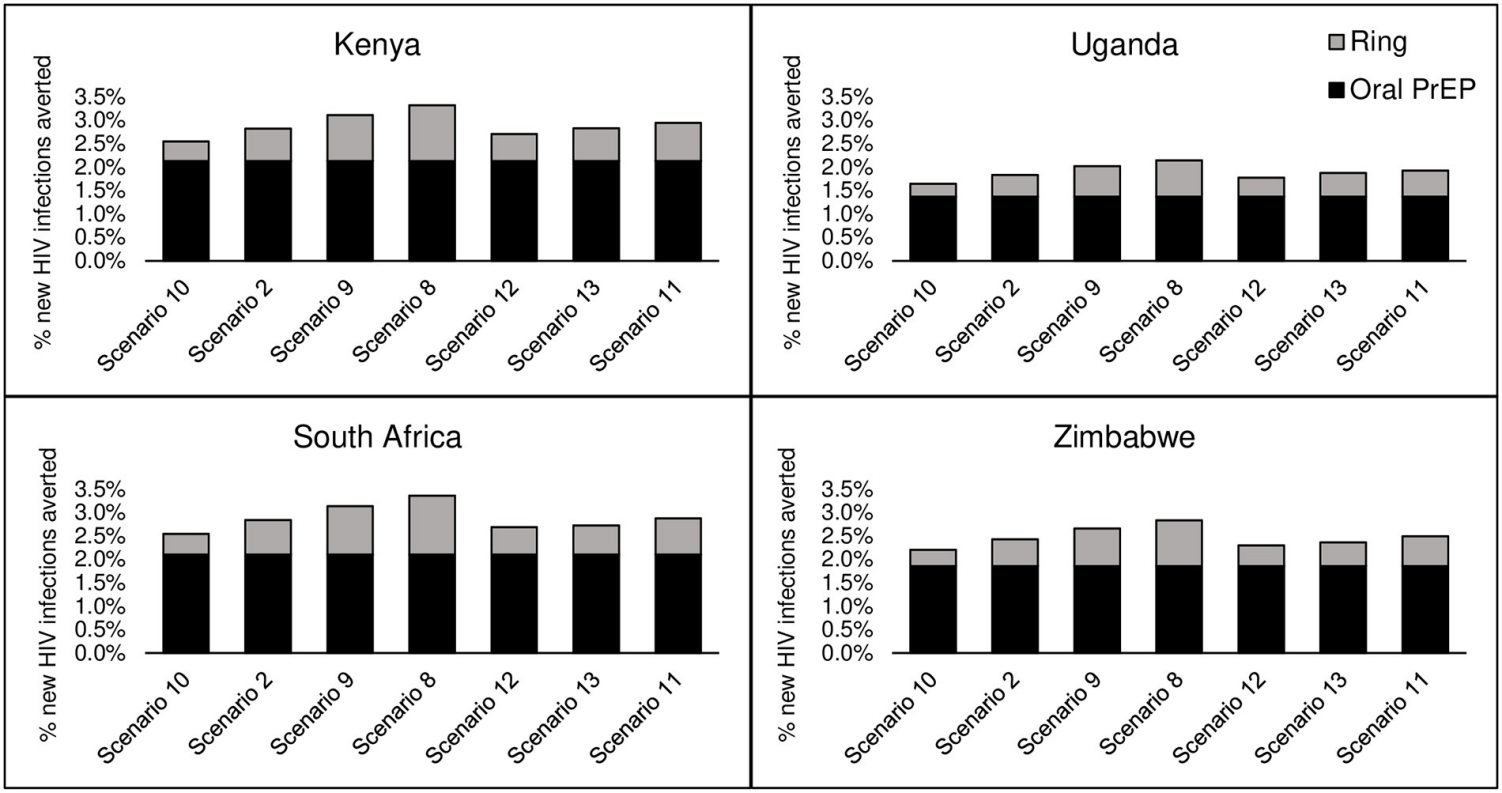
Impact of ring effectiveness scenarios on the percentage of new HIV infections averted by PrEP (oral plus ring) (2018–2035). Scenarios: (10) Low ring effectiveness, (2) Moderate ring effectiveness, (9) High ring effectiveness, (8) Ambitious ring effectiveness, (12) lower ring adherence among younger women, and (13) both higher ring adherence among FSWs and lower adherence among younger women, (11) higher ring adherence among FSWs.

HIV infections averted are directly correlated with the level of ring effectiveness used in the scenario, ranging from 30% effectiveness on the low end to 85% effectiveness on the high end. Fig 3 presents the scenarios that vary effectiveness overall and by risk group. These are compared with the moderate-effectiveness scenario (50% effectiveness), which is the same moderate scenario (Scenario 2) seen in the previous three research questions (i.e., What is the range of potential impact of the dapivirine ring plus oral PrEP in the most extreme scenarios? How will different scenarios for coverage of the dapivirine ring affect the impact of the HIV prevention program? How will different scenarios for ART scale-up affect dapivirine ring impact?), where there is moderate coverage of both oral PrEP and the ring and ART scale-up assumes achievement of 90-90-90 by 2020. This illustrates the results of reduced adherence in younger women, the combination of higher adherence among FSWs and lower adherence among younger women, and finally, higher adherence among FSWs. The patterns are similar across countries, with some variation in levels. For example, in South Africa, given the relative incidence rates in the risk groups included in the model, reduced adherence among younger women has a greater impact on new HIV infections than increased adherence among FSWs. This parallels the results seen when increasing ring coverage in FSWs or reducing coverage in younger women.

### How does the cost-effectiveness of the dapivirine ring vary in these scenarios?

**Fig 4:**
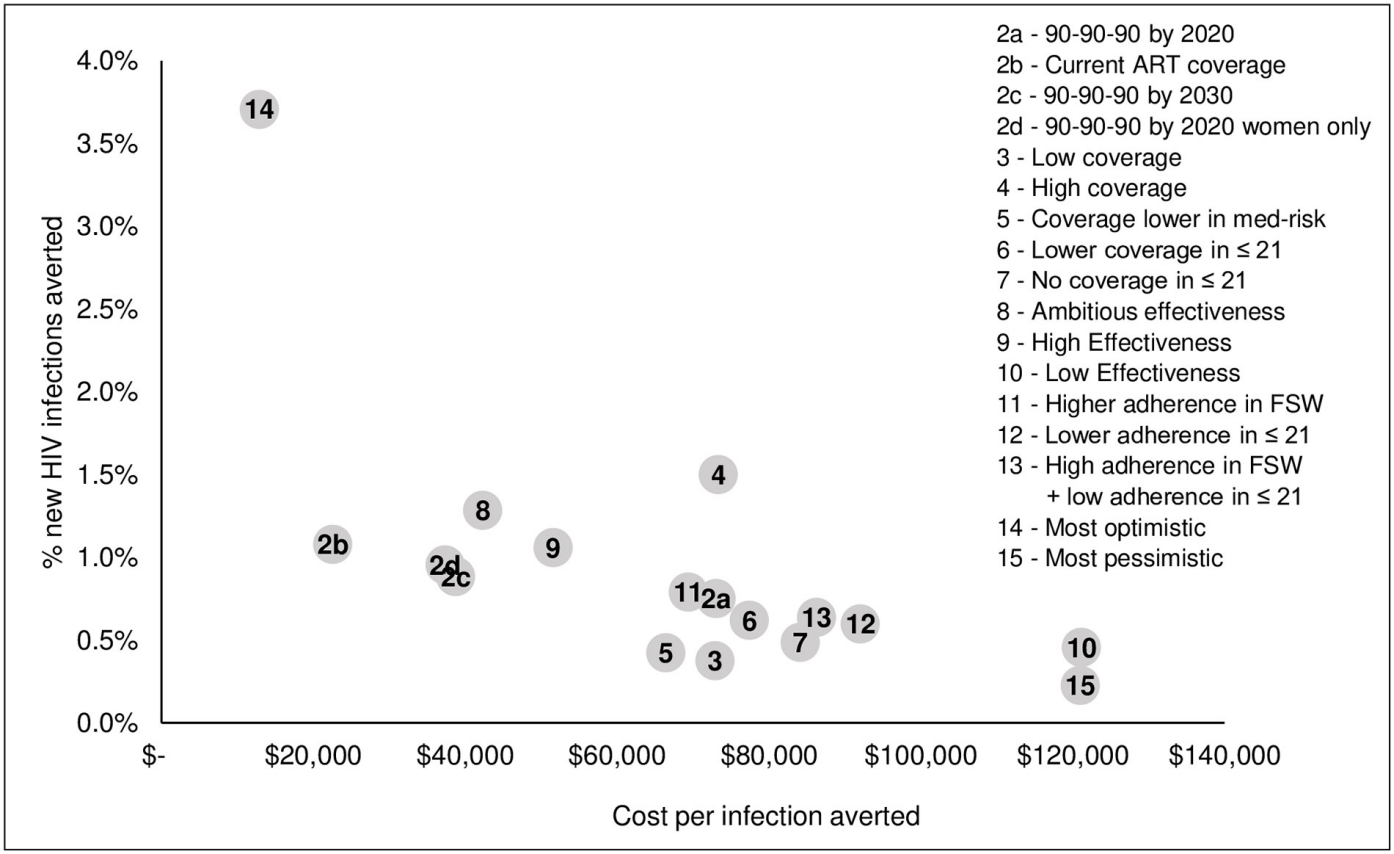
Incremental cost per HIV infection averted by use of the dapivirine ring in South Africa (2018–2035).

The cost per HIV infection averted varies widely with the assumptions made about ring effectiveness and ART scale-up. Fig 4 illustrates the range for South Africa, where the cost per HIV infection averted ranges from US$13,000 to US$121,000 in the highest- and lowest-impact scenarios, respectively. The x-axis shows the incremental cost per HIV infection averted, while the y-axis shows the percent of new HIV infections averted, relative to a counterfactual of moderate oral PrEP and no ring use (Scenario 1).

For the moderate scenario in South Africa, assuming achievement of 90-90-90 by 2020 (Scenario 2a), the cost per infection averted by the ring is US$73,000, compared with US$40,000 per infection averted by oral PrEP with no ring and achievement of 90-90-90 by 2020 (Scenario 1a), and US$13,000 for oral PrEP with no ring when continuation of current ART coverage is assumed (Scenario 1b) and US$23,000 for the ring under this same ART scenario (Scenario 2b). S2 Table shows the cost per HIV infection averted by ring for all countries and scenarios.

## Discussion

Just as total contraception use has been shown to increase (and unintended pregnancies decrease) with the number of contraceptive methods made available [3], we anticipate the addition of dapivirine ring to the choice of prevention options could result in 11% to 132% more impact for ARV-based prophylaxis than oral PrEP alone. Our analysis showed how the impact of the dapivirine ring depends on the level of ring coverage achieved in each target population and the effectiveness of the ring, which can be increased by increasing adherence. It also depends on the level of scale-up of other interventions, such as ART and oral PrEP. Not surprisingly, the maximum potential impact in absolute terms is far greater in South Africa than in any other country evaluated, with 310,000 new HIV infections averted from 2018–2035, while the potential impact across the four countries is lowest in Zimbabwe (8,000). In relative terms, the greatest potential impact is seen in Kenya, with 7.7% of new HIV infections averted, while the lowest potential impact is in Uganda (4.5%). As expected, with higher ring coverage and consistent use, the impact of the ring increases. During implementation of the ring, maximizing adherence would increase both impact and cost-effectiveness of the intervention. The scenarios presented here include modest assumptions on uptake, based on feedback from the stakeholder consultation, in the absence of implementation research. Focusing on uptake would increase the overall impact of the intervention, but that would not in itself increase cost-effectiveness.

We have assumed here that the ring is less effective than oral PrEP; however, the effectiveness and uptake of both products may vary by subpopulation, based on these groups’ different needs and product preferences. A recent user preference study among product-experienced (with placebo injections, tablets, and rings) participants emphasized the importance of offering a variety of method options in order to meet the heterogenous needs and preferences of different women [19,20]. It is also unknown whether the effectiveness of oral PrEP is actually higher than that of the ring in real-world settings. Because the ring is more user-independent than oral PrEP (requiring only monthly placement of the ring rather than daily use of a pill), there is the potential for the effectiveness of the ring to be greater than oral PrEP in real-world application. This may have significant implications for potential future uptake, as prevention efficacy is a strong determinant of method choice [20].

The impact and cost-effectiveness results depend on the likelihood of infection for the users. Any HIV prevention intervention will be more impactful and cost-effective among populations with higher incidence. With high ART coverage, the likelihood of HIV transmission—and infection—decreases, and therefore the additional impact of the ring and other primary prevention interventions would also decrease. For example, if South Africa were to achieve 90-90-90 by 2020, incidence would decline by more than half from 2015 to 2020. This would be a welcome scenario, as lowering HIV infections is the overall goal, but it also would mean that every prevention intervention will be twice as expensive per infection averted, if evaluated as a single intervention. In a scenario in which the current pace of ART scale-up remains steady or slows (Scenarios b-d), as UNAIDS is anticipating [21], the ring’s potential impact would be higher, as would that of other primary HIV prevention interventions.

The numbers for cost per HIV infection averted that are presented in our findings may seem high. Readers should keep in mind that the scenarios presented here assume not only scale-up of oral PrEP, but also in most cases scale-up of ART to the 90-90-90 targets. Because both of these interventions serve to decrease HIV incidence in the population, the incremental impact of yet another prevention intervention will be smaller than if the ring had been introduced at a time prior to massive scale-up of ART and in the absence of oral PrEP. At this point in the HIV epidemic response, additional interventions are still needed to fill gaps left by scale-up of ART and other prevention approaches, but the incremental cost-effectiveness of these gap-filling interventions, especially those that require delivery on an ongoing basis, such as any form of PrEP, will necessarily be high. Moreover, if we compare the cost per HIV infection averted by ART to that of primary prevention, we need to consider the fact that ART for the index case reduces the risk to all partners. Primary prevention, on the other hand, must overcome the probability of meeting an infected partner first.

These results are more robust in illustrating relative cost-effectiveness for different possible scenarios within countries than in cross-country comparison. As few country-specific costing studies of oral PrEP have been conducted, and none have been conducted for the ring, to date, the unit costs for these interventions, and the resulting cost-effectiveness findings, should be interpreted with caution. Unit costs may vary by implementation model, and costs of both oral PrEP and the ring may come down with improved implementation and efficiencies of scale. Additionally, the cost per person-year of treatment was held constant over time. If the actual cost of treatment were to decrease relative to the cost of ring delivery, then the ring would become less cost-effective. Conversely, if the cost to deliver the ring in any country were lower than the cost used in this analysis, then the ring would become more cost-effective. The differences between countries are affected not just by the cost of the intervention and of ART, but also by the HIV incidence in the population that is being provided with the intervention.

It is worth calling attention to the fact that this analysis did not include any scenarios in which low-risk women used the dapivirine ring, meaning that the uptake assumptions, even in the highest coverage scenarios, are quite conservative. While PrEP is generally discussed as being needed for high risk populations [2], lower risk women in generalized epidemic situations may also wish to protect themselves from HIV, and some may find the ring particularly attractive due to its discretion, ease of use, and lack of side effects compared with oral PrEP, particularly if it is formulated in combination with contraception to protect against unwanted pregnancy [20]. Uptake among lower risk women in generalized epidemics could increase the impact of the ring above the levels projected in this analysis.

With the paucity of data to inform every parameter used in the modeling, the limitations to this analysis are significant. While we set our ranges based on early study results and expert consultation, uptake, cost, and effectiveness of both the ring and oral PrEP when fully implemented could be very different. In addition, while the ring and oral PrEP were provided to FSWs and medium risk women in our analysis, it should be noted that the medium risk group in the model is a poor proxy for women at elevated risk for HIV, as this group is not well characterized in the real world. Little is known about how to identify women at elevated risk in implementation settings, how big this subpopulation is, and the degree to which their risk is increased. The levels of impact and cost-effectiveness in this modeling exercise should be interpreted with these caveats in mind.

## Conclusion

Given the persistently high rates of HIV infection among women despite the scale-up of ART and VMMC, and the importance of product choice for effective use, new HIV prevention methods for women are needed. Depending on a number of factors explored in this paper, the dapivirine vaginal ring may provide additional impact on control of the HIV epidemic. Greater understanding of the real-world cost and potential uptake of the intervention would improve our ability to estimate its possible impact and cost-effectiveness. However, ultimately, the purpose of the ring is to increase uptake of HIV prevention to prevent HIV acquisition, not necessarily to maximize cost-effectiveness.

Because the ring is a new delivery modality, there are many unknowns. Implementation research and demonstration and pilot projects are needed to improve our understanding of the ring’s potential impact and to devise strategies to maximize it. This modeling exercise offers a wide range of scenarios that incorporate the considerable uncertainty about ring uptake, consistency of use, and effectiveness, as well as oral PrEP and HIV treatment use over the next two decades. Even amid this uncertainty, however, it is clear that for the ring to have the greatest impact, implementers and donors should invest in maximizing uptake and adherence to the ring among women who are in need of HIV prevention and who are unlikely to consistently use other primary prevention interventions.

## Supporting information

S1 Table Dapivirine ring and Oral PrEP scenarios. (XLSX)

S2 Table Impact and cost-effectiveness of the dapivirine ring 2018–2035. (XLSX)

S3 Table Person-years of PrEP in each scenario (2018–2035). (XLSX)
